# Excision of 5-hydroxymethyluracil and 5-carboxylcytosine by the thymine DNA glycosylase domain: its structural basis and implications for active DNA demethylation

**DOI:** 10.1093/nar/gks845

**Published:** 2012-09-08

**Authors:** Hideharu Hashimoto, Samuel Hong, Ashok S. Bhagwat, Xing Zhang, Xiaodong Cheng

**Affiliations:** ^1^Department of Biochemistry, Emory University School of Medicine, 1510 Clifton Road, Atlanta, GA 30322, ^2^Molecular and Systems Pharmacology graduate program, Emory University School of Medicine, 1510 Clifton Road, Atlanta, GA 30322 and ^3^Department of Chemistry, Wayne State University, 443 Chemistry, Detroit, MI 48202, USA

## Abstract

The mammalian thymine DNA glycosylase (TDG) is implicated in active DNA demethylation via the base excision repair pathway. TDG excises the mismatched base from G:X mismatches, where X is uracil, thymine or 5-hydroxymethyluracil (5hmU). These are, respectively, the deamination products of cytosine, 5-methylcytosine (5mC) and 5-hydroxymethylcytosine (5hmC). In addition, TDG excises the Tet protein products 5-formylcytosine (5fC) and 5-carboxylcytosine (5caC) but not 5hmC and 5mC, when paired with a guanine. Here we present a post-reactive complex structure of the human TDG domain with a 28-base pair DNA containing a G:5hmU mismatch. TDG flips the target nucleotide from the double-stranded DNA, cleaves the *N*-glycosidic bond and leaves the C1′ hydrolyzed abasic sugar in the flipped state. The cleaved 5hmU base remains in a binding pocket of the enzyme. TDG allows hydrogen-bonding interactions to both T/U-based (5hmU) and C-based (5caC) modifications, thus enabling its activity on a wider range of substrates. We further show that the TDG catalytic domain has higher activity for 5caC at a lower pH (5.5) as compared to the activities at higher pH (7.5 and 8.0) and that the structurally related *Escherichia coli* mismatch uracil glycosylase can excise 5caC as well. We discuss several possible mechanisms, including the amino-imino tautomerization of the substrate base that may explain how TDG discriminates against 5hmC and 5mC.

## INTRODUCTION

Mammalian DNA cytosine modification is a dynamic process and occurs by converting cytosine (C) to 5-methylcytosine (5mC), established by specific DNA methyltransferases, and then to 5-hydroxymethylcytosine (5hmC or H) by ten-eleven-translocation (Tet) proteins (1–4). Tet proteins can further oxidize 5hmC to 5-formylcytosine (5fC) and 5-carboxylcytosine (5caC) (5,6). The genomic 5fC and 5caC contents are very low [5–10 fmol (5)] compared to hundreds of pmols of 5hmC present in many tissues and cell types examined (1). In addition, the content of 5-hydroxymethyluracil (5hmU), the deamination product of 5hmC, is also relatively low [<3.5 pmol (1)]. These data suggest that modification products of 5hmC are either produced rarely or are short-lived possibly because of removal by subsequent enzymatic reactions.

**Figure 1.**
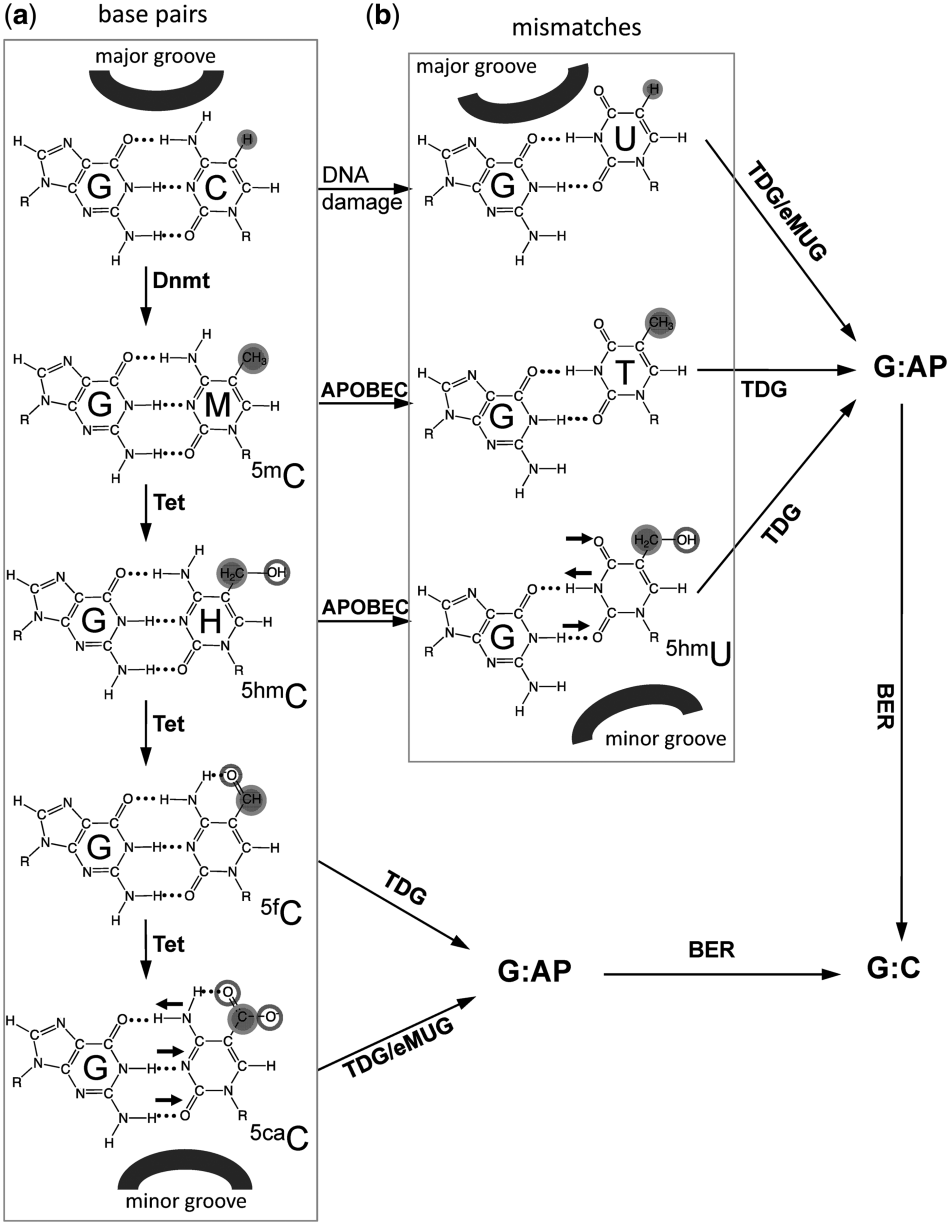
A putative pathway of DNA demethylation involving DNA methylation by DNMTs, hydroxylation by Tet proteins, deamination by members of APOBEC superfamily, and base excision by TDG linked to base excision repair (BER). In addition, eMUG can excise 5caC as well (see Figure 2). DNA major groove and minor groove sides are indicated. Horizontal small arrows indicate the hydrogen bond donors and acceptors for 5caC and 5hmU bases. (**a**) C, 5mC and its oxidized derivatives (5hmC, 5fC and 5caC) form base pairs with an opposite G. (**b**) Deamination-linked mismatches.

The mammalian thymine DNA glycosylase (TDG) has been proposed to be involved in active DNA demethylation through the removal of deamination products of 5mC or its oxidized derivatives by the base excision repair pathway (7–9). Consistent with this role, the activation-induced deaminase (AID), a DNA-cytosine deaminase, is reported to be required to demethylate pluripotency genes during reprogramming of the somatic genome in embryonic stem cell fusions (10), and AID-deficient animals are less efficient in erasure of DNA methylation in primordial germ cells (11). Additionally, another member of the AID/APOBEC family (12), APOBEC3A, is more efficient at 5mC deamination than AID (13). High expression of a member of the AID/APOBEC family may promote 5mC deamination, creating a T:G mismatch (14,15), or 5hmC deamination, producing a 5hmU:G mismatch (8), which would be subject to excision by TDG (9,16) (Figure 1).

Also of particular interest are recent reports that TDG can excise 5fC and 5caC (but not 5hmC and 5mC) from DNA (6,17). This new specificity of TDG suggests a deamination-independent active DNA demethylation pathway through Tet-mediated oxidation of 5hmC (Figure 1). Here, we explore the structural and biochemical basis of TDG excision of 5hmU (a deamination product) and 5caC (a Tet-mediated oxidation production).

Human TDG catalytic domain (residues 111–308) has been crystallized with an abasic analog (tetrahydrofuran) within a 22-bp DNA with one 3′-overhanging adenine or thymine (22+1 bp) (18). We initially followed this published crystallization procedure (18) and collected a complete dataset at 4.0 Å resolution of the catalytic mutant N140A in complex with the 22+1-bp DNA containing a G:5caC site (Supplementary Figure S1). The diffraction quality of these type of P6_5_ crystals (containing two TDG molecules and one DNA duplex) varied significantly and required screening of many crystals to achieve ∼3.0 Å resolution (18). During the course of the study, structures were reported for the same TDG fragment in complex with the same 22+1-bp DNA containing either an A:5caC mismatch or a modified 5caC (with a 2′-fluoro substitution on the deoxyribose of 5caC) paired with G (19). Both crystals diffracted asymmetrically to 3 Å along the *a* and *b* axes and 4 Å along the *c* axis (19).

Here we focus on the structural study of the TDG domain in complex with DNA containing a G:5hmU mismatch and compare the structure to that of TDG with a 2′-deoxy-2′-fluoroarabinouridine (20). We also investigate the biochemical properties of TDG on the G:5caC substrate and compare them to that of a TDG-related mismatch-specific uracil glycosylase (MUG) from *Escherichia coli*.

## MATERIALS AND METHODS

### Expression and purification of TDG

Human TDG residues 111-308 (pXC1056) and its mutants (see below) were expressed using the pET28b vector as described (18). The proteins were expressed in *E. coli* BL21(DE3)-Gold cells with the RIL-Codon plus plasmid (Stratagene). Cultures were grown at 37°C until the OD_600_ reached 0.5; at that point the temperature was shifted to 16°C, and isopropyl β-D-1-thiogalactopyranoside (IPTG) was added to 0.4 mM to induce expression. Cells were re-suspended with a 4× volume of 300 mM NaCl, 20 mM sodium phosphate, pH 7.4, 20 mM imidazole, 1 mM dithiothreitol (DTT) and 0.25 mM phenylmethylsulphonyl fluoride and sonicated for 5 min (1 s on and 2 s off). The lysate was clarified by centrifugation twice at 38 000 g for 30 min. Hexahistidine fusion protein was isolated on a nickel-charged chelating column (GE Healthcare). The His_6_ tag was removed by adding 50 Units of thrombin to the imidazole eluate from the Ni column and incubated for 16 h at 4°C, leaving six extraneous N-terminal amino acids (GSHMAS). The cleaved protein was further purified by collection of flow through of a HiTrap SP column (GE-Healthcare) and concentrated. The concentrated protein was then loaded onto a Superdex 75 (16/60) column (equilibrated with 100 mM NaCl, 20 mM HEPES, pH 7.0, 1 mM DTT) where it eluted as a single peak corresponding to a monomeric protein. The purification of eMUG has been described previously (21).

### Mutagenesis

The following mutants were generated by PCR mutagenesis, expressed and purified similar to the wild-type protein: single point mutations of N140A (pXC1057), N140D (pXC1105), A145S (pXC1156), N157A (pXC1155), S200A (pXC1112), K201A (pXC1120), N230D (pXC1113), S271A (pXC1114), S271H (pXC1122) and quadruple mutant of P198-G199-S200-K201 to AAAA (pXC1123).

### Crystallography of TDG and DNA complexes

For co-crystallization with the 28-bp DNA, 0.35 mM of TDG wild-type protein was mixed with 0.2 mM of annealed oligonucleotide (synthesized by the New England Biolabs, Inc.): 5′-CAG CTC TGT A**CG** TGA GCG ATG GAC AGC T-3′ and 5′-AGC TGT CCA TCG CTC A**XG** TAC AGA GCT G-3′ where X is 5hmU. The 5-hydroxymethyl-deoxyU phosphoramidites used for DNA synthesis were purchased from Glen Research. Crystals appeared within 24 h under the conditions of 30% polyethylene glycol (PEG) 4000, 0.2 M ammonium acetate, 0.1 M sodium acetate, pH 4.6. Crystals were cryoprotected by soaking in mother liquor supplemented with 20% ethylene glycol. X-ray diffraction datasets were collected at the SER-CAT beamline (22ID-D) at the Advanced Photon Source, Argonne National Laboratory and processed using HKL2000 (22). The structures were solved by molecular replacement by PHENIX (23) using TDG and the abasic DNA complex structure [PDB 2RBA (18)] as the search model. Electron density for DNA was easily interpretable, and its model was built using the programs O and Coot (24). PHENIX refinement scripts were used for refinement, and the statistics shown in Supplementary Table 1 were calculated for the entire resolution range. The *R*_free_ and *R*_work_ values were calculated for 5% (randomly selected) and 95%, respectively, of observed reflections.

### DNA glycosylase activity assay

TDG activity assays were performed using various oligonucleotides labeled with 6-carboxy-fluorescein (FAM) and monitoring the excision of the target base by denaturing gel electrophoresis following NaOH hydrolysis of the abasic site (Supplementary Figure S2). TDG protein (0.5 µM) and an equal amount of double-stranded FAM-labeled 32-bp duplexes were mixed in 20 µL nicking buffer (10 mM Tris-HCl, pH 8.0, 1 mM EDTA, 0.1% BSA) and incubated at 37°C for 30 min: (FAM)-5′-TCG GAT GTT GTG GGT CAG **X**GC ATG ATA GTG TA-3′ (where **X** = C, 5mC, 5hmC, U, T, 5hmU or 5caC) and 5′-TAC ACT ATC ATG CGC TGA CCC ACA ACA TCC GA-3′.

The reactions were stopped by adding 2 µL of 1 N NaOH and by boiling for 10 min. Twenty microliters of loading buffer (98% formamide, 1 mM EDTA and 1 mg/ml of Bromophenol Blue and Xylene Cyanole) were added, and the reaction mixtures were boiled for another 10 min. Samples were immediately cooled in ice water and loaded onto a 10×10 cm^2^ 15% denaturing gel containing 7 M urea, 24% formamide, 15% acrylamide and 1× Tris-Borate-EDTA (TBE). The gels were run in 1× TBE buffer for 60 min at 200 V. FAM-labeled single-stranded DNA was visualized under UV exposure.

For enzymatic reactions under single turnover condition, the FAM-labeled 32-bp duplexes (250 nM) and 10-fold excess of TDG catalytic domain or eMUG (2.5 µM) were incubated for 0–30 min (for G:5caC at 37°C or room temperature) or 0–5 min (for G:U at 4°C) in 0.1% BSA and 1 mM EDTA buffered by a mixture of 15 mM citric acid, 30 mM BisTris-propane and 15 mM (cyclohexylamino)ethanesulfonic acid (CHES) adjusted to the indicated pH. The intensities of the FAM-labeled DNA were determined by Typhoon Trio+ (GE Healthcare) and quantified by the image-processing program ImageJ (NIH). The data were fitted to nonlinear regression using software GraphPad PRISM 5.0d (GraphPad Software Inc.): [Product] = *P*_max_(1 − *e*^−^*^kt^*), where *P*_max_ is the product plateau level, *k* is the observed rate constant and *t* is the reaction time.

### DNA binding assay

TDG protein (1.0 µM) and 0.5 µM of 32-bp-FAM labeled DNA (same as above) were mixed in 20-µL nicking buffer (10 mM Tris-HCl, pH 8.0, 1 mM EDTA, 0.1% BSA) and incubated at 37°C for 15 min. Samples were loaded onto a 10 × 10 cm^2^ 10% native polyacrylamide gel in 1× Tris-Borate-EDTA (TBE) buffer and ran for 40 min at 100 V.

## RESULTS

### TDG forms a stable complex with specific DNA

**Figure 2.**
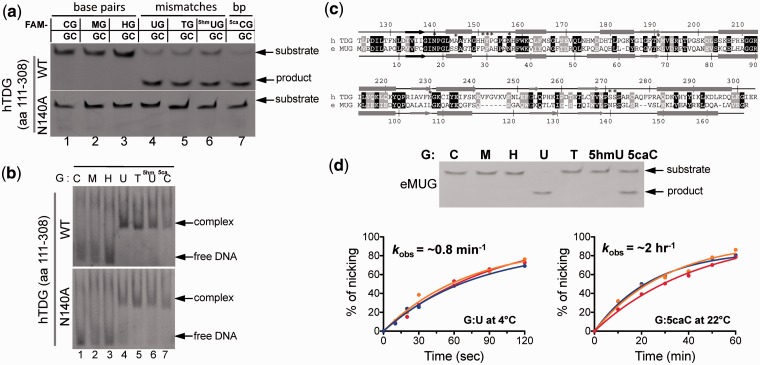
Base excision and binding activities of TDG catalytic domain in the context of a double-stranded CpG dinucleotide. (**a**) Double-stranded 32-bp oligonucleotides bearing a single CpG dinucleotide were incubated with equal amount of the glycosylase domain of TDG or its noncatalytic mutant N140A at 37°C for 30 min. The oligonucleotide was labeled with FAM on the top strand, and the modification status was indicated (M = 5mC and H = 5hmC). The products of the reactions were separated on a denaturing polyacrylamide gel, and the FAM-labeled strand was excited by UV and photographed. (**b**) DNA binding assays were performed by incubating 0.5 µM FAM-labeled oligonucleotides with 1 µM of TDG at 37°C for 15 min. (**c**) Pairwise sequence alignment of human TDG domain (top line) and *E. coli* MUG (bottom line). Secondary structural elements are shown above or below the aligned sequences. White-on-black residues are invariant between the two sequences examined, while gray-highlighted positions are conserved (R and K, E and D, Q and N, T and S, F, Y and W, V, I, L and M, and G and P). Positions highlighted by * are active site residues responsible for catalysis (Asn140) and/or proposed for substrate base recognition (only two of them, Asn140 and Asn157, are invariant between human TDG and *E. coli* MUG). (**d**) eMUG is active on G:U and G:5caC substrates (top panel). Reactions were performed at room temperature (approximately 22°C) for 30 min with [E_eMUG_] = [S_DNA_] = 5 µM. The kinetic activities of eMUG on G:U substrates at 4°C (bottom left panel) and G:5caC at room temperature (approximately 22°C) (bottom right panel) were measured under single turnover condition ([E_eMUG_] = 2.5 µM and [S_DNA_] = 0.25 µM) at three different pH values (5.5 in red, 7.5 in orange and 8.0 in blue curves).

We first measured the glycosylase and binding activities of the TDG fragment (residues 111–308) using various 32-base-pair (bp) DNA oligonucleotides, each containing a single modified base X (X = C, M, H, U, T, 5hmU or 5caC) within a G:X pair in a CpG sequence. As expected, glycosylase activity was not observed with TDG on oligonucleotides bearing the ‘natural’ G:C, G:M and G:H base pairs, while substrates bearing G:T, G:U and G:5hmU mismatches were efficiently cleaved (Figure 2a). Therefore, TDG is capable of acting on deamination generated products. In addition, TDG is capable of excising 5caC when paired with a guanine, which presumably preserves Watson-Crick base-pair hydrogen bonds (Figure 1a). A catalytic mutant (N140A) is inactive on all substrates under the conditions of pH 8.0 at 37°C for 30 min (Figure 2a), as reported previously (25,26). The glycosylase activity correlates well with the ability to form a specific complex in electrophoretic mobility-shift assay (Figure 2b), under the condition of 2:1 molar ratio of enzyme to DNA.

*Escherichia coli* mismatch uracil glycosylase (eMUG) is related to human TDG both in sequence (27) (31% identity and 43% similarity; Figure 2c) and in structure (28). Under the same single turnover conditions used for TDG, eMUG excises G:5caC in addition to G:U mismatch (Figure 2d), albeit much more slowly. We did not detect eMUG activity on oligonucleotides bearing G:T and G:5hmU mismatches, in agreement with a previous report that eMUG has 10^4^–10^5^ fold reduced activity on T and 5hmU compared to U (29).

### Crystallization of TDG catalytic domain bound with 28-bp DNA

**Figure 3.**
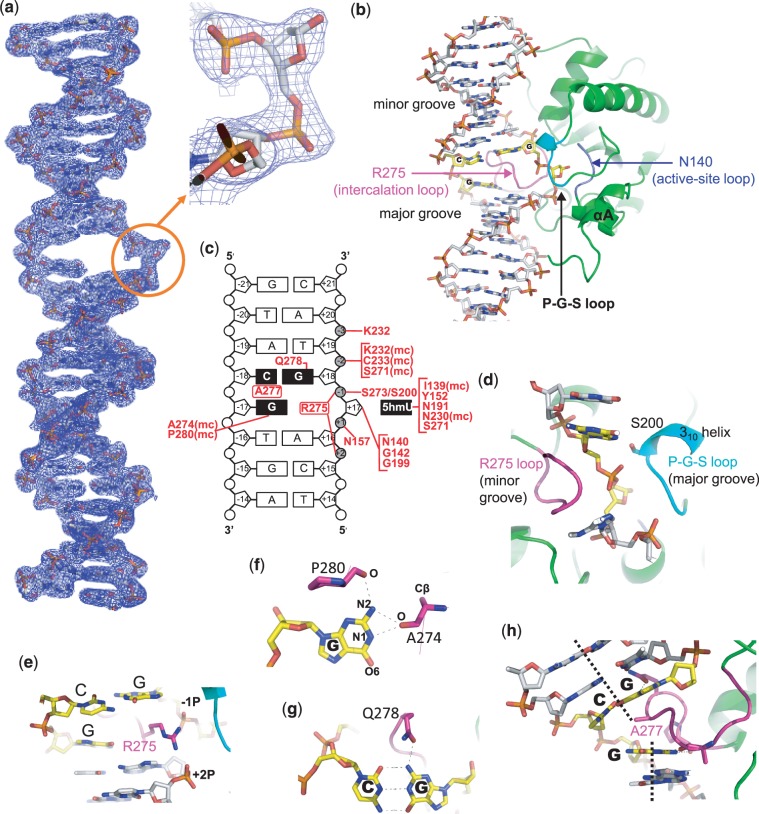
Structure of TDG in complex with G:5hmU containing DNA. (**a**) 2Fo-Fc electron density, contoured at 1*σ* above the mean, for the entire 28-bp DNA used in the TDG structure determination. The insert is an enlarged abasic sugar with a hydrolyzed C1′. (**b**) Overall structure of the WT TDG complex. DNA is in stick model, and TDG is in ribbon model. The Arg275-containing loop is colored in magenta, the P-G-S loop is in cyan and catalytic loop in blue. (**c**) Summary of the TDG–DNA interactions: mc, main-chain-atom-mediated contacts; black boxes represent the CpG sequence and extrahelical 5hmU. (**d**) The Arg275-containing intercalation loop (in magenta) and the P-G-S loop (in cyan) approach the modified DNA strand from opposite directions. (**e**) Arg275 penetrates into the DNA helix from the minor groove. (**f**) The three hydrogen bonds formed with the intrahelical orphaned guanine. (**g**) Gln278 forms a hydrogen bond from the minor groove side with Gua of the adjoining G:C base pair. (**h**) Ala277 intercalates between the central Cyt and Gua of the unmodified strand.

To improve the resolution of X-ray diffraction, we varied the lengths of oligonucleotides used for crystallization. Only after we used a 28-bp oligonucleotide were we able to consistently grow crystals that formed in space group C2 and which reached the modest resolutions of 2.3 to 2.6 Angstroms. We report here the TDG structure in complex with the 28-bp DNA containing a G:5hmU mismatch at 2.5 Å resolution (Supplementary Table S1). We observed electron density for TDG residues 111–305 and all 28 base pairs of DNA (Figure 3a and Supplementary Figure S3). TDG flips the target nucleotide 5hmU from the double-stranded DNA, cleaves the *N*-glycosidic bond and leaves the abasic sugar in the flipped state. The cleaved 5hmU base remains in a binding pocket of TDG. We will first describe the overall structure and then the detailed interactions involving 5hmU.

### Overall structure of the 1:1 TDG–DNA complex

Unlike the structure of TDG with the 22+1-bp DNA that consisted of two protein molecules per DNA (18), our crystals with the 28-bp DNA contain a single protein-DNA complex at 1:1 ratio per crystallographic asymmetric unit (even under crystallization conditions of ∼2:1 ratio of protein to DNA), confirming that the functional reaction complex involves a TDG monomer (30). The protein component is highly similar to that of the previous structure (18), with a root mean squared deviation of ∼0.6 Å when comparing the 182 pairs of Cα atoms (residues 123–304). One significant difference is that despite the fact that we used the same length protein, our new structure revealed an additional N-terminal helix (αA; residues 115–122), which extends toward the DNA minor groove together with the N-terminal tail (Figure 3b).

The majority of the protein–DNA interactions in the two structures are also very similar, except for the flipped out target base in our new structures. Briefly, TDG makes phosphate contacts spanning five base pairs but mostly on the phosphates surrounding the modified nucleotide (two 5′- and three 3′-phosphate groups; summarized in Figure 3c). The backbone of the DNA strand containing the modified base is firmly gripped by the loop containing Arg275 and residues P198-G199-S200 followed by a 3_10_ helix, approaching from opposite directions (major and minor grooves, respectively) (Figure 3d). The side chain of Arg275 penetrates into the DNA helix from the minor groove, occupying the space left by the flipped-out modified nucleotide (Figure 3e). The positively charged guanidino group of Arg275 electrostatically interacts with the phosphate group immediately 3′ to the modified base, and the 5′-phosphate two bases away (−1 and +2 phosphate groups in Figure 3c).

An ‘intercalation’ loop containing Arg275 (amino acids 270–281) also contains residues interacting with both guanines of the CpG dinucleotide. The intrahelical orphaned guanine hydrogen bonds with the main chain carbonyl oxygen atoms of Ala274 and Pro280 (Figure 3f). The exocyclic N2 atom of the guanine of the neighboring G:C pair hydrogen bonds with Gln278 from the minor groove (Figure 3g). This TDG–guanine interaction ensures the specificity of base excision to be within the CpG dinucleotide (18). No interaction to the neighboring G:C pair in the major groove side is observed, suggesting that the modification status at C5 of the Cyt in the complementary strand (methyl, hydroxymethyl or carboxyl) would have no impact on TDG activity (31). In addition, Ala277 intercalates between the Cyt and Gua bases of the unmodified strand, resulting in an ∼25° kink (Figure 3h).

### Structure of a post-reactive complex: TDG with a cleaved 5hmU base

**Figure 4.**
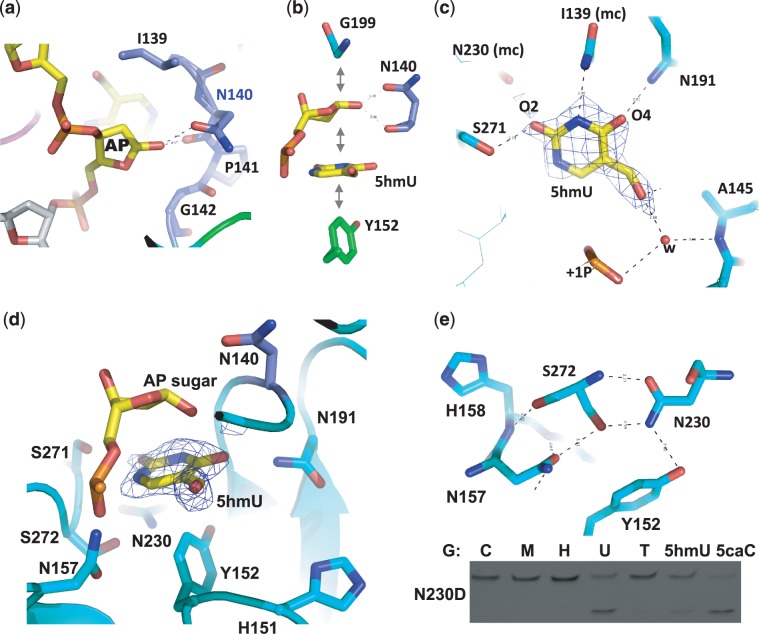
The binding of 5hmU in the active site. (**a**) In the WT structure, the *N*-glycosidic bond of the extrahelical nucleotide is cleaved, and a hydroxyl oxygen atom has been attached to the C1′ of the sugar ring (see Figure 3a inset). (**b**) Favorable face-to-face and edge-to-face hydrophobic interactions between the sugar, the cleaved 5hmU base and Tyr152. (**c**) Omit electron density, contoured at 3.5*σ* above the mean, is shown for omitting 5hmU. The hydrogen bond interactions (dashed lines) with the polar atoms of 5hmU are within 3.0 Å distance cutoff: mc, main-chain-atom-mediated contacts. (**d**) The 5hmU-binding pocket is rich in polar atoms. (**e**) A hydrogen-bonding network involves both side chain and main chain atoms of depicted residues. The activity of N230D is shown.

In the TDG structure complexed with G:5hmU pair, the *N*-glycosidic bond of the modified nucleotide is cleaved leaving the abasic sugar bound in the active site surrounded by Ile139, Gly199, Asn140 and Gly142 (Figure 4a and b). There is an extra electron density near the C1′ atom indicating the existence of an attached hydroxyl oxygen (Figure 3a insert) and suggesting that the hydroxylation already occurred (see below). The catalytic residue Asn140 directly approaches the sugar ring with its main-chain and side-chain carbonyl oxygen atoms interacting with the hydroxyl oxygen attached to the C1′ of the abasic sugar ring. Similar to the structures of uracil DNA glycosylase (32,33), the cleaved base of 5hmU remains bound in a cage-like pocket via hydrophobic interactions that involve stacking face to face with the abasic sugar ring and face to edge with the aromatic ring of Tyr152 (Figure 4b), suggesting a tight binding of the abasic reaction product (26,30,34–36). Such tight binding by the glycosylase (which lacks AP lyase activity) protects the abasic site from nonspecific processing until subsequent repair activities are recruited to the lesion site and TDG sumoylation facilitates enzymatic turnover (37).

The Watson–Crick polar edge of the cleaved 5hmU base is involved in interactions with the side-chain hydroxyl oxygen atom of Ser271 and the main-chain carbonyl oxygen atom of Asn230 (via the O2 atom), the main-chain amide nitrogen atom of Ile139 (via the N3 atom) and the side-chain amino group of Asn191 (via the O4 atom) (Figure 4c). The hydroxyl oxygen atom of 5hmU interacts directly with the main-chain amide nitrogen of Gly142 (Supplementary Figure S2c) and water-mediated contacts with Ala145 and the 5′-phosphate group of the abasic site (Figure 4c). We note that the interaction between Ile139 and the ring N3 atom does not form an ideal hydrogen bond because both the amide nitrogen atom and the N3 atom under normal physiological conditions carry a hydrogen atom.

The fact that TDG retains its cleaved base in a cage-like pocket is due to the lack of an open cleft through which the cleaved base can diffuse out to solvent. Since human TDG has a broad substrate spectrum for base modifications opposite guanine (16,17,38), we reasoned that the cage should be able to accommodate a variety of cleaved bases via the stacking interactions between the abasic sugar and Tyr152 (a conserved residues among members of TDG family; Supplementary Figure S4), whereas the interactions with Ser271 and Asn230 (to O2), Ile139 (to N3) and Asn191 (to N4), in the cage are potentially applicable to C-based modifications, for example, 5caC (Supplementary Figure S2d). We also note that residues whose side chains point to the cage, Ser271, Ser272, His151, Asn157, Asn191 and Asn230, can all act as either hydrogen bond donor and/or acceptor (Figure 4d), allowing flexibility in accommodating a variety of bases. On the other hand, a network of polar interactions (Figure 4e) is critical for stabilizing the active site, including Tyr152 (stacking with the target base), Asn230 (interacting with O2), Ser272 (whose main chain and side chain polar atoms are saturated with interactions) and Asn157 (interacting with 5′-phosphate of the flipped nucleotide). Mutation of Asn230 to its corresponding aspartate (N230D) produced a mutant with greatly reduced activity. Whereas G:U and G:5caC were still processed, the mutant has residual activity on G:5hmU and lost the catalytic activity on G:T substrates (Figure 4e).

### Structural comparison to a pre-reaction complex

**Figure 5.**
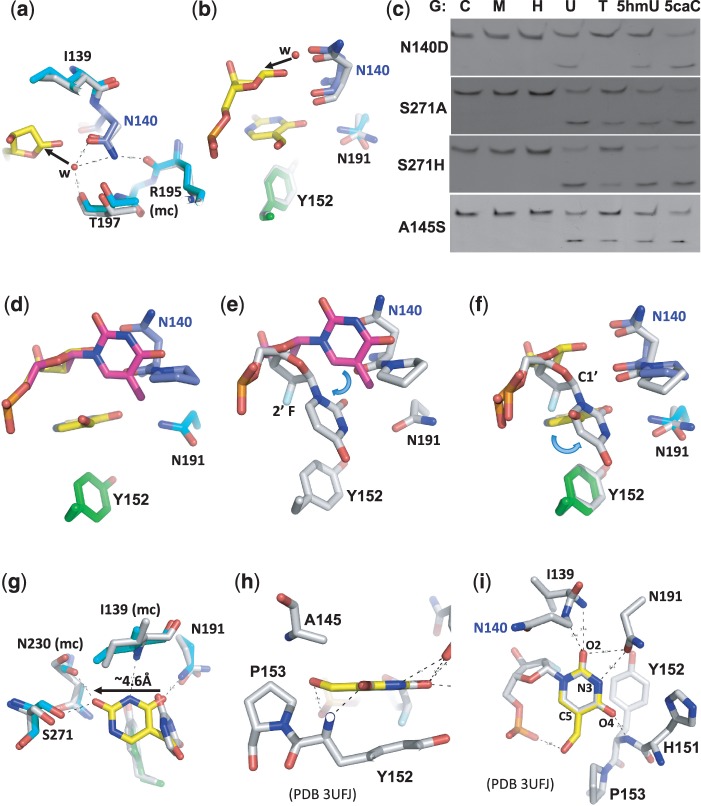
Comparison of post- and pre-reactive complex structures. (**a**, **b**) Superimposition of the post-reactive complex (in color) and the pre-reactive complex [in gray; PDB 3UFJ (20)] shows a putative nucleophilic water molecule, held in position by the side chain carbonyl oxygen atom of Asn140 in the pre-reactive complex, attacks the C1′ from the opposite position of the leaving base, generating the C1′-hydrolyzed abasic sugar as shown in the post-reactive complex (in yellow). (**c**) The activities of TDG mutants N140D, S271A, S271H and A145S. (**d, e**) Superimposition of a normal intrahelical thymine (colored in magenta) onto the post-reactive complex (panel d) or the pre-reactive complex (panel e) suggests a bent relative to the sugar ring and a rotation around the glycosidic bond. (**f**, **g**) Superimposition of the post-reactive complex (in color) and the pre-reactive complex (in gray) suggests the base undergoes another rotation after cleavage (panel f) and moves towards Ser271 (panel g). (**h, i**) Superimposition of a 5hmU base (in yellow) onto the flipped uracil in the pre-reactive complex (PDB 3UFJ).

We compared our post-reaction complex structure with that of a pre-reactive complex containing 2′-deoxy-2′-fluoroarabinouridine, a mimic of deoxyuridine that is not cleaved by TDG (20). One of the key observations revealed by the pre-reaction complex was a putative nucleophilic water molecule, held in position by the side-chain carbonyl oxygen atom of Asn140 and the backbone carbonyl oxygen of Thr197 (20) (Figure 5a). No such water molecule was found in the corresponding position of the post-reactive complex where the C1′ hydroxylation already occurred (Figure 3a insert). Superimposition of the pre- and post-reaction complex structures revealed the attack trajectory by the water molecule at the C1′ occurs from the opposite side of the leaving base (Figure 5b). Other structurally characterized DNA *N*-glycosylases have an acidic residue in the active site coordinating the proposed nucleophilic water molecule [Asp145 of human uracil DNA glycosylase (39) and Asp144 of *Bacillus stearothermophilus* MutY (40)]. We mutated Asn140 of TDG to the corresponding carboxylate (N140D), reasoning that the attack of water could benefit from the base catalysis provided by the carboxylate group of N140D. However, N140D only has measurable but reduced activity on G:U, G:5hmU and G:5caC and no detectable activity on G:T substrate (Figure 5c). N140D probably disrupts the hydrogen bond between the side-chain amino group of Asn140 and the backbone carbonyl oxygen atom of Arg195 (Figure 5a) and thus destabilizes the catalytic loop conformation.

Superimpositions of a normal intrahelical thymine with the post- and pre-reactive complexes, respectively (Figure 5d and e), suggest that the everted yet uncleaved base rotates around the glycosidic bond as well as bends relative to the sugar ring. Once the N1–C1′ glycosidic bond is cleaved, the base is further rotated (Figure 5f) and moved ∼4.6 Å to the position in the post-reactive complex where the O2 interacts with Ser271 (Figure 5g). The corresponding position of Ser271 is an asparagine in eMUG (Figure 2c) or a histidine in the UDG superfamily, including human uracil DNA glycosylase (hUNG) (41) (Supplementary Figure S5a). Since eMUG, like TDG, has activity on 5caC (Figure 2d), whereas hUNG exhibited no activity toward 5caC (6), we mutated Ser271 to histidine (S271H) in addition to alanine (S271A). However, both mutations do not affect substrate specificity (Figure 5c), probably because the Ser271–O2 interaction occurs only after the cleavage.

Finally, we superimposed a 5hmU base onto the flipped uracil base in the pre-reactive complex (Figure 5h and i). The 5-hydroxymethyl group could fit the space between Ala145 and Pro153 (Figure 5h) with the hydroxyl oxygen interacting with one of the 5′-phosphate oxygen atoms (Figure 5i). We mutated Ala145 to serine (A145S) because the corresponding residue in eMUG is a serine (Figure 2c). The side chain of Ser23 of eMUG is directed toward the 5-position of the flipped uracil, and it was suggested that the Ser23 would lower the efficiency of thymine excision (with a methyl group at the 5-position) but not prevent it (42). However, the TDG A145S mutation does not affect substrate specificity (Figure 5c), in agreement with a previous mutational analysis that A145S mutation has no effect on processing G:T substrate (26). This is probably because the A145S side-chain hydroxyl oxygen could rotate away from the substrate to accommodate various modifications at the 5-position.

Interestingly, in the pre-reactive TDG complex, the exocyclic O2 oxygen atom of the flipped uracil base is ∼3.5 Å away from the main-chain amide nitrogen atoms of Ile139 and Asn140 as well as the side-chain carbonyl oxygen atom of Asn191 (20) (Figure 5i). The Asn191 side-chain carbonyl oxygen atom is also ∼3.5 Å from the proton-bearing ring N3 atom (20). The N191A mutation causes decreased TDG activities for G:U and G:T (20). We note that a simple rotation around the side-chain χ_2_ torsion angle of Asn191 would allow the side-chain amino group to form a hydrogen bond with the un-protonated N3 atom of Cyt (or its derivatives) (Supplementary Figure S1). Furthermore, the interaction of exocyclic O4 of uracil with the main-chain amide nitrogen of Tyr152 (20) (Figure 5i) is converted to the interaction of exocyclic N4 (NH_2_) of 5caC with the side chain of His151 (19) (Supplementary Figure S1b). Thus, the TDG active site provides interactions to the polar edges of a uracil (and its derivatives 5hmU and thymine) as well as 5caC (all substrates of TDG).

### The effect of *N*-glycosidic bond stability on catalysis

The rather tolerant nature of the TDG active site raises the question as to why 5hmC is not a substrate for TDG while 5caC is a substrate. If we suppose that 5hmC was flipped into the active site similarly to 5caC, it would have identical interactions with TDG along the Watson–Crick edge, and perhaps one hydrogen bond less than 5caC at the C5 position. Such a small difference seems unlikely to be the sole reason why 5hmC is not a substrate and, moreover, 5hmU is a substrate despite the presence of C5 substitution (Figure 2a).

**Figure 6.**
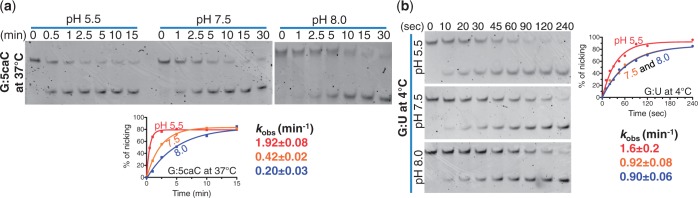
The activity of TDG catalytic domain as a function of pH. The activity of TDG catalytic domain on (**a**) G:5caC at 37°C and (**b**) G:U substrates at 4°C under single turnover condition ([E_TDG_] = 2.5 µM and [S_DNA_] = 0.25 µM) at three different pH values. The reaction on G:U substrate was measured at 4°C because the reaction was complete within 1 min at 37°C or room temperature (∼22°C) (data not shown).

Rather than selective base recognition or an inability of TDG to completely flip 5hmC (or 5mC or even C) into its active site, an alternative explanation has been suggested for the specificity of TDG that is attributed to the reactivity of the *N*-glycosidic bond (17,43) as estimated by electronic substituent constant (*σ*_m_) (44) of the C5 substituent. TDG has greater activity for C (and U) analogs with an electron-withdrawing C5 substituent (*σ*_m_ > 0), such that TDG can rapidly excise 5fC (with an *σ*_m_ value of 0.35) but is not active against 5mC (with an *σ*_m_ value of −0.07) and 5hmC (with *σ*_m_ = 0) (17). Interestingly, the *σ*_m_ value for the deprotonated state (COO^−^) of 5caC is −0.10 (even lower than that of 5mC) but 0.37 for the protonated form (COOH)(44). Although the robust TDG activity on 5caC at pH 7.5 (17) or pH 8.0 (Figure 1a) is not predicted, given that the carboxylate group should be deprotonated at those pH values, yielding a negative *σ*_m_ value, we investigated whether lowering the pH would enhance TDG activity on 5caC as a result of increasing protonation of the carboxylate group. TDG activity on the G:U substrate is known to be relatively constant for pH 5.5–9 (45). Accordingly, we measured TDG activities on both G:U and G:5caC substrates under single turnover conditions (i.e. [E_TDG_] >> [S_DNA_]) at three different pH values of 5.5, 7.5 and 8.0. To eliminate the effect of buffer on activity, we used a mixture of citric acid, 1,3-bis(tris(hydroxymethyl)methylamino)propane (Bis-tris propane) and 2-CHES that was adjusted to each pH. We find that the TDG catalytic domain has higher activity (by a factor of 5 and 9.5, respectively) for the G:5caC at pH 5.5 (*k*_obs_ = 1.9 min^−1^), as compared to the activities at pH 7.5 (*k*_obs_ = 0.4 min^−1^) and 8.0 (*k*_obs_ = 0.2 min^−1^) (Figure 6a). Although the activity for the G:U substrate is also higher at pH 5.5 (by approximately a factor of 2) than that at higher pH (45) (Figure 6b), the more dramatic enhancement of activity on G:5caC at lower pH suggests that the chemical nature of the target nucleotide, as predicted by the *σ*_m _value of the 5-position substituent, can contribute to TDG being active on 5caC, but that alone does not fully account for the activity at neutral or higher pH. Other, more specific, interactions with the target base are likely involved, particularly knowing the fact that a family of the plant DNA glycosylases is capable of excising 5mC (46) and 5hmC (Supplementary Figure S6), albeit slowly (47).

## DISCUSSION

### Impact of base pairing and glycosidic torsion angles on catalysis

Several factors may affect the ability of the target nucleotide being flipped from the intrahelical to a fully extrahelical position in the active site. One factor to consider is whether various oxidation states of 5mC derivatives perturb the stability of DNA duplex differently, as base-pairing dynamics have a critical role in allowing hUNG to capture a spontaneously extruded lesion (41). The melting temperatures (*T*_m_) of oligonucleotides containing G:C, G:5mC, G:5hmC, G:5fC or G:5caC are within 1°C of each other, suggesting no significant differences among the base-paring properties of 5mC derivatives (48,49). The hydrogen bond energy of the Watson–Crick-type base pair of G:5caC was theoretically estimated to be −30 kcal/mol, while the naturally occurring G:C base pair shows the hydrogen bond energy of −27 kcal/mol (50), suggesting that the modified base pair was more stable than the canonical base pair by 3 kcal/mol.

The second possible factor to consider is whether oxidized 5mC derivatives affect the DNA backbone conformation, as MutM distinguishes a target 8-oxoG from G via the sugar pucker conformation in the DNA backbone (51). This happens because the 8-oxo substitution causes a steric clash with the sugar while rotating the glycosidic bond torsion angle. However, the 5-position substituents would not cause steric clash with sugar while rotating the glycosidic bond. In summary, while the structure of the substrate provides some clues to the substrate specificity of TDG, it does not fully explain the lack of activity against 5hmC.

### An interrogation along the multi-step flipping pathway

Extensive studies on DNA glycosylase enzymes, such as hUNG and 8-oxoguanine DNA glycosylases (hOGG and bacterial MutM) [reviewed in (39,52)], showed that they recognize damaged bases through a multi-step interrogation process. The enzymes distort DNA by bending it followed by intrahelical interrogation to detect a lesion, flipping of potential substrate nucleotides to varying degrees and rejection of non-substrate nucleotide back to DNA helices, allowing only a true substrate to reach the active site. Our DNA binding data show that the TDG catalytic domain binds significantly more weakly to C, 5mC and 5hmC than to substrate bases including 5caC, suggesting a discrimination step before stable (perhaps flipped) complex formation (Figure 2b). In the TDG–DNA post-reactive complex examined here, we did not observe any protein side-chain interaction in the major groove of DNA where the modifications at the C5 position are positioned. However, two side chains of TDG, Lys201 of the 3_10_ helix (whose side chain density is disordered) and Ser200 of the P-G-S loop (whose side chain forms an interaction with the 3′-phosphate group of the flipped nucleotide; Figure 3c) are located in the major groove and could potentially form interactions with C5 modifications (Figure 3d) while scanning along the DNA major groove via an active intrahelical interrogation and extrusion mechanism as proposed for MutM (51). In addition, the P-G-S loop of TDG can be superimposed well with the corresponding loop of hUNG involved in examination of a partially flipped thymine in the search for uracil in DNA (41) (Supplementary Figure S5b and c). However, the TDG mutants of S200A, K201A or PGSK to AAAA neither affect TDG substrate specificity nor activity under the conditions tested (Supplementary Figure S5d). These results suggest that the P-G-S loop is unlikely to play a strong role in discriminating between different 5-substituents.

### The potential effect of amino/imino tautomeric forms on catalysis

**Figure 7.**
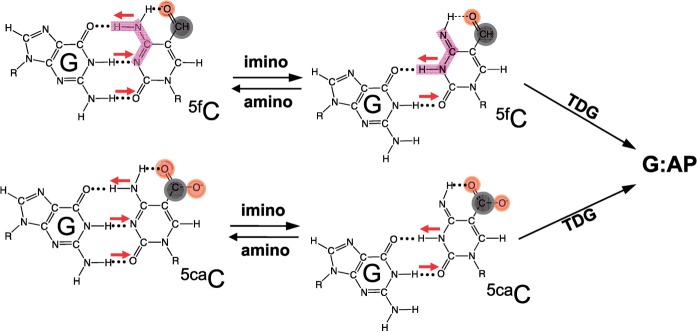
Amino-imino tautomerization. 5fC and 5caC exhibit an intramolecular hydrogen bond that could shift the amino/imino equilibrium toward the imino tautomeric form which would then base pair with guanine in a mismatch-like wobble pattern.

A strong intramolecular hydrogen bond has been observed between the exocyclic N4 amino group and the carbonyl oxygen at C5 of 5fC in the free nucleoside form (49,53). It was hypothesized that the existence of such a hydrogen bond would shift the amino-imino equilibrium (54,55), which would enable 5fC to form two, instead of three, hydrogen bonds with an opposite G (Figure 7), equivalent to a G:T or G:5hmU ‘wobble’ pair (Figure 1b). Previously observed mutagenic potential of 5fC in cells (54,56) suggested the possible existence of the imino tautomeric form, which could result in the mutagenic incorporation of an adenine opposite of the 5fC during DNA replication. Indeed a small amount (1–2%) of adenine incorporation was observed in DNA polymerase reactions *in vitro* (49,55). In a more recent study, the discrimination of GTP over ATP is reduced by a factor of ∼30 for 5fC template in comparison with C template during *in vitro* RNA polymerase II transcription (57).

We speculate that TDG might take advantage of the tendency of G:5fC and G:5caC, both of which contain a 5-position carbonyl oxygen, to form a mismatch-like wobble hydrogen bonding pattern (Figure 7) and turn them into substrates. Thus, the common theme of TDG substrates could be the ability to form wobble pairs. This wobble geometry might be important for TDG in the initial recognition of the substrate pair. After flipping, TDG is able to hold a variety of bases in the active site pocket. The enzyme–DNA complex seen in the gel retardation assay (Figure 2b) could be either the substrate in the wobble pairing conformation or the product complex (E•P) not the initial substrate complex (E•S), which is a possible reason that there is no gel shift with 5hmC and 5mC. Additionally, the observed increase in the reaction rate at pH 5.5 (Figure 6a) is consistent with increased protonation of N3 required for tautomerization. Finally, in light of the fact that plant ROS1 is capable of excision of both 5mC and 5hmC bases when paired with a guanine (Supplementary Figure S6), the mechanism of substrate recognition by this glycosylase must be different from that of TDG and eMUG. This difference in the two enzyme families has yet to be studied structurally, (bio)chemically and thermodynamically.

## ACCESSION NUMBERS

Protein Data Bank: The coordinates and structure factors of the post-reactive complex of human TDG domain with 5hmU have been deposited with accession number 4FNC.

## SUPPLEMENTARY DATA

Supplementary Data are available at NAR Online: Supplementary Table 1 and Supplementary Figures 1–6.

## FUNDING

U.S. National Institutes of Health (GM049245-18 to X.C. and GM057200-09 to A.S.B.); Georgia Research Alliance Eminent Scholar (to X.C.) and the Department of Biochemistry at the Emory University School of Medicine (use of the Southeast Regional Collaborative Access Team synchrotron beamlines at the Advanced Photon Source of Argonne National Laboratory). Funding for open access charge: NIH.

*Conflict of interest statement*. None declared.

## Supplementary Material

Supplementary Data
