# Supplementary material for: Excision of 5-hydroxymethyluracil and 5-carboxylcytosine by the thymine DNA glycosylase domain: its structural basis and implications for active DNA demethylation
Source: Nucleic Acids Res. 2012 Sep 8;40(20):10203–14. doi: 10.1093/nar/gks845 (PMC3488261; doi:10.1093/nar/gks845)
Supplement: Supplementary Data [file supp_40_20_10203__index.html]

Excision of 5-hydroxymethyluracil and 5-carboxylcytosine by the thymine DNA glycosylase domain: its structural basis and implications for active DNA demethylation — Excision of 5-hydroxymethyluracil and 5-carboxylcytosine by the thymine DNA glycosylase domain: its structural basis and implications for active DNA demethylation — Supplementary Data 

# Excision of 5-hydroxymethyluracil and 5-carboxylcytosine by the thymine DNA glycosylase domain: its structural basis and implications for active DNA demethylation

## Supplementary Data

files

**Files in this Data Supplement:**

- Supplementary Data - pdf file
