# Supplementary material for: Excision of 5-hydroxymethyluracil and 5-carboxylcytosine by the thymine DNA glycosylase domain: its structural basis and implications for active DNA demethylation
Source: Nucleic Acids Res. 2012 Sep 8;40(20):10203–14. doi: 10.1093/nar/gks845 (PMC3488261; doi:10.1093/nar/gks845)
Supplement: Supplementary Data [file supp_gks845_nar-01592-m-2012-File005.pdf]

Supplementary Data

Excision of 5-hydroxymethyluracil and 5-carboxylcytosine by the TDG glycosylase domain: its structural basis and implications for active DNA demethylation

Hideharu Hashimoto[1], Samuel Hong[1,2], Ashok S. Bhagwat[3], Xing Zhang[1] and Xiaodong Cheng[1]

[1]Departments of Biochemistry, [2]Molecular and Systems Pharmacology graduate program, Emory University School of Medicine, 1510 Clifton Road, Atlanta, GA 30322, USA

[3]Department of Chemistry, Wayne State University, 443 Chemistry, 5101 Cass Avenue, Detroit, MI 48202, USA

Correspondence should be addressed to:

Xiaodong Cheng (xcheng@emory.edu; phone: 404-727-8491; fax: 404-727-3746)

Email addresses for all authors:

Hideharu Hashimoto (hhashi3@emory.edu)

Samuel Hong (shong27@emory.edu)

Ashok S. Bhagwat (axb@chem.wayne.edu)

Xing Zhang (xzhan02@emory.edu)

Xiaodong Cheng (xcheng@emory.edu)

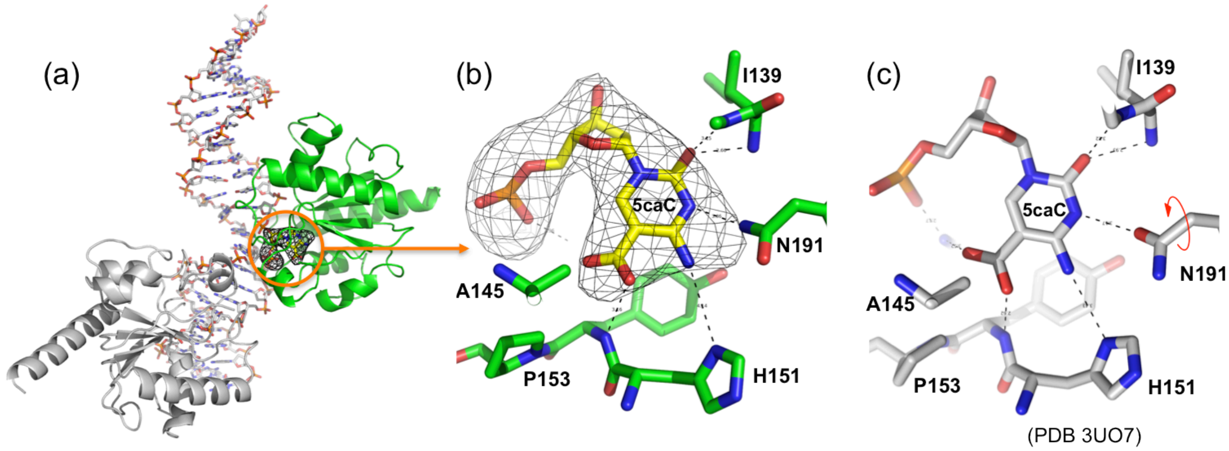

Supplementary Figure S1. A low-resolution structure of the TDG N140A mutant in complex with 5caC containing 22+1 bp DNA

(a) For co-crystallization with the 22-bp DNA plus one 3' overhanging adenine or thymine, 5'-CAG CTC TGT A**CG** TGA GCG ATG GA-3' and 5'-CCA TCG CTC A**XG** TAC AGA GCT GT-3' where X=5caC, N140A mutant TDG (0.35 mM) was mixed with 0.2 mM of annealed oligonucleotide and the crystals grew under the conditions of 20% polyethylene glycol (PEG) 3350, 0.2 M sodium fluoride (pH ~7.5-8.0). The rock-like crystals appeared in 24 h and formed in space group $P6_5$ with cell dimensions of a=b=162 Å and c=56 Å. Crystals were cyroprotected by soaking in mother liquor supplemented with 20% ethylene glycol. A low-resolution data set at 4.0 Å resolution were collected at the beamline 22ID-D of the Advanced Photon Source at the Argonne National Laboratory. The $P6_5$ structure contains two TDG molecules (green and grey) and one DNA molecule (in stick model).

(b) Omit electron density map at 4.0 Å resolution, contoured at 3.5σ above the mean, is shown for omitting 5caC. The residues surrounding the 5caC are shown.

(c) The active site adopted from PDB 3UO7, a structure determined by the diffraction patterns exhibited strong anisotropy between 3 and 4 Å resolutions (17). We suggest that rotating the side chain χ2 torsion angle of Asn191 would allow the side chain amino group to form a hydrogen bond with the proton-deficit N3 atom of 5caC.

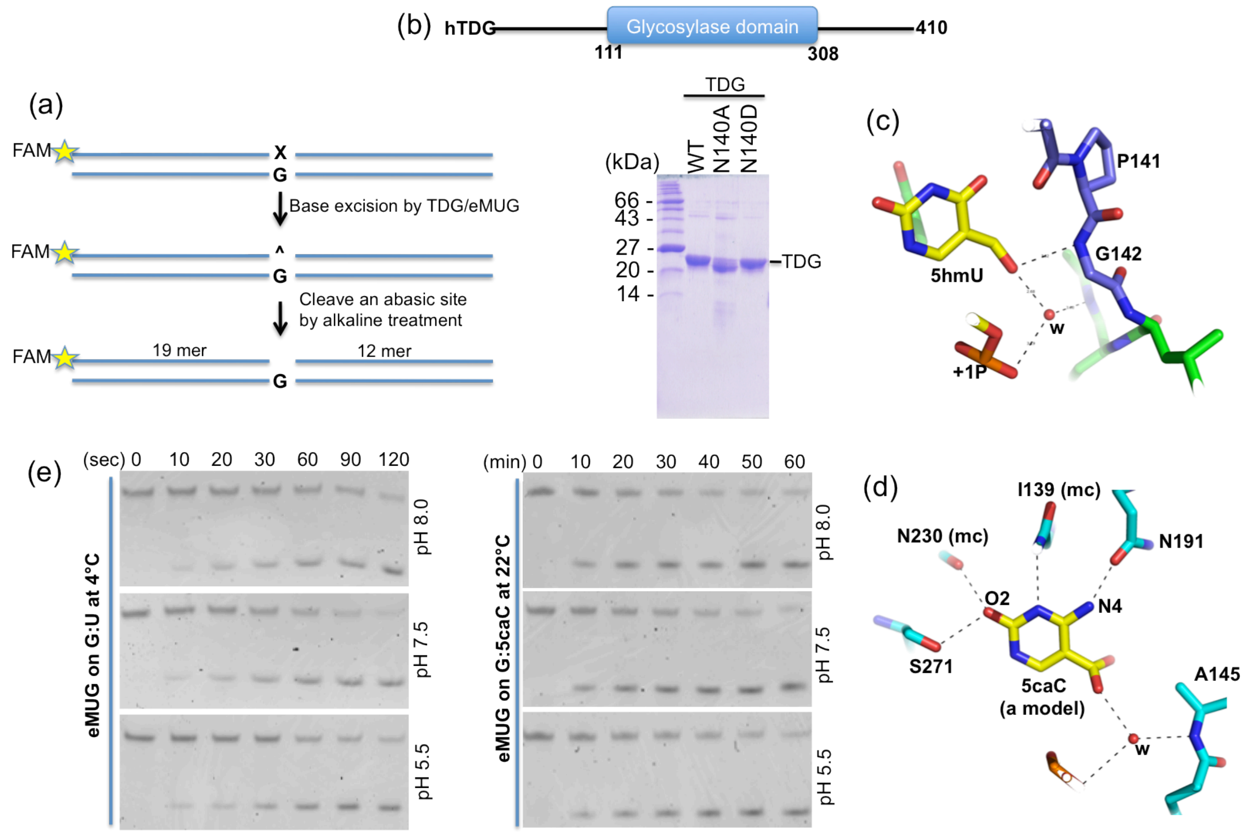

Supplementary Figure S2. Purified TDG glycosylase domain

(a) Overview of the glycosylase activity assay. (b) Schematic representation of TDG (top panel) and SDS-PAGE gel showed the purified recombinant TDG wild type and mutants used in this study (bottom panel).

(c) The hydroxyl group of 5hmU forms a hydrogen bond with the main chain amide nitrogen of Gly142 (in blue) and water-mediated contacts with Ala145 (in green and further away from the reader) and the 5’ phosphate group of the abasic site.

(d) A 5caC base can be modeled into the same binding site with an optimum hydrogen bond between the main chain amide nitrogen atom (NH) and the proton-deficit ring N3 atom. Rotating the side chain $\chi 2$ torsion angle of Asn191 would allow the side chain carbonyl oxygen atom to form a hydrogen bond with the N4 amino group (NH_2) of 5caC.

(e) The activity of eMUG on G:U substrates at 4 °C (left panel) and G:5caC at room temperature (approximately 22 °C) (right panel) under single turnover condition ($[E_{TDG}]$=2.5 μM and $[S_{DNA}]$=0.25 μM) at three different pH values.

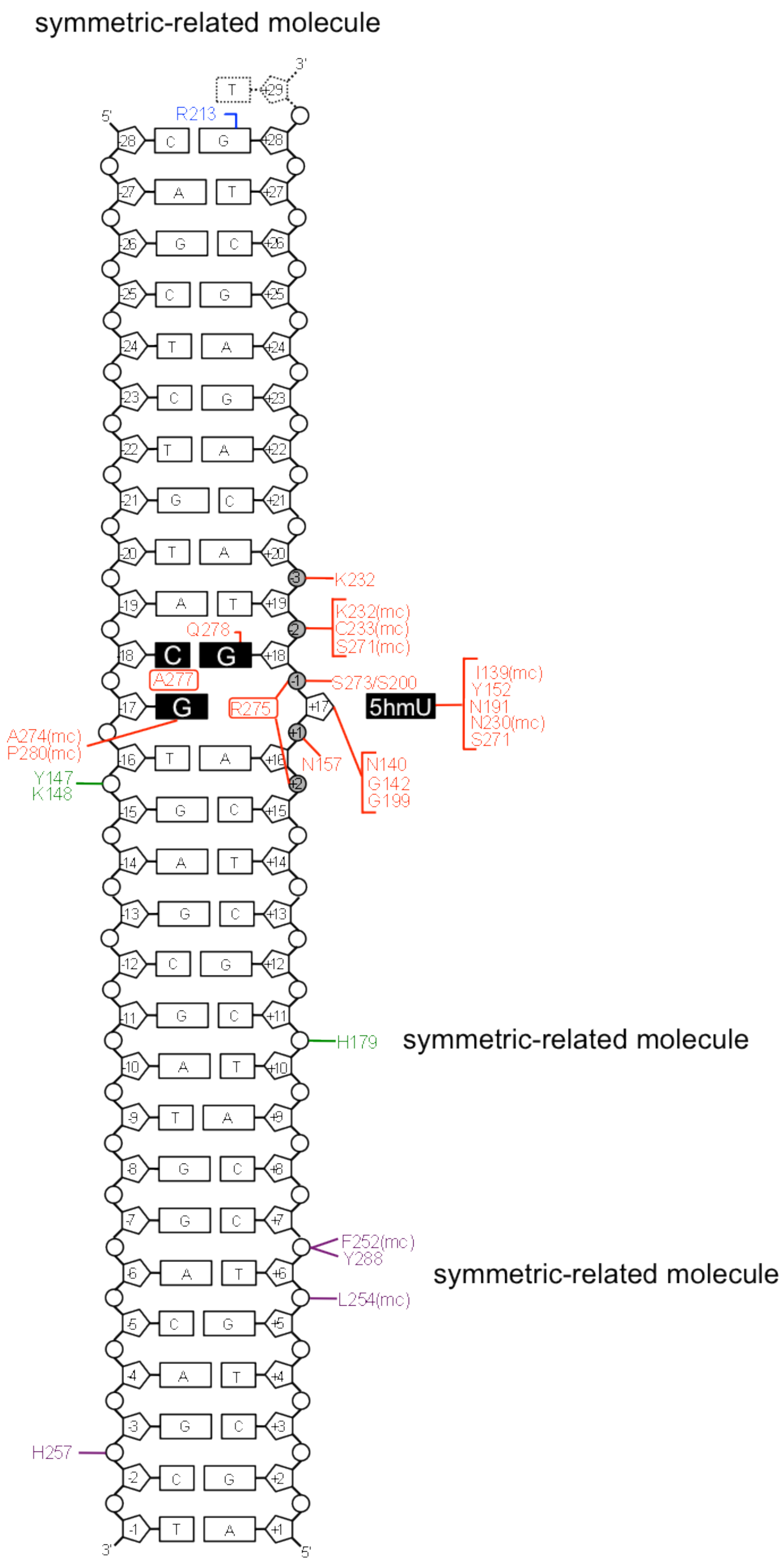

Supplementary Figure S3. Schematic TDG-DNA interactions. The 28 bp DNA molecule is contacted specifically with one TDG molecule (colored in red), and nonspecifically to three crystallographic symmetry-related molecules.

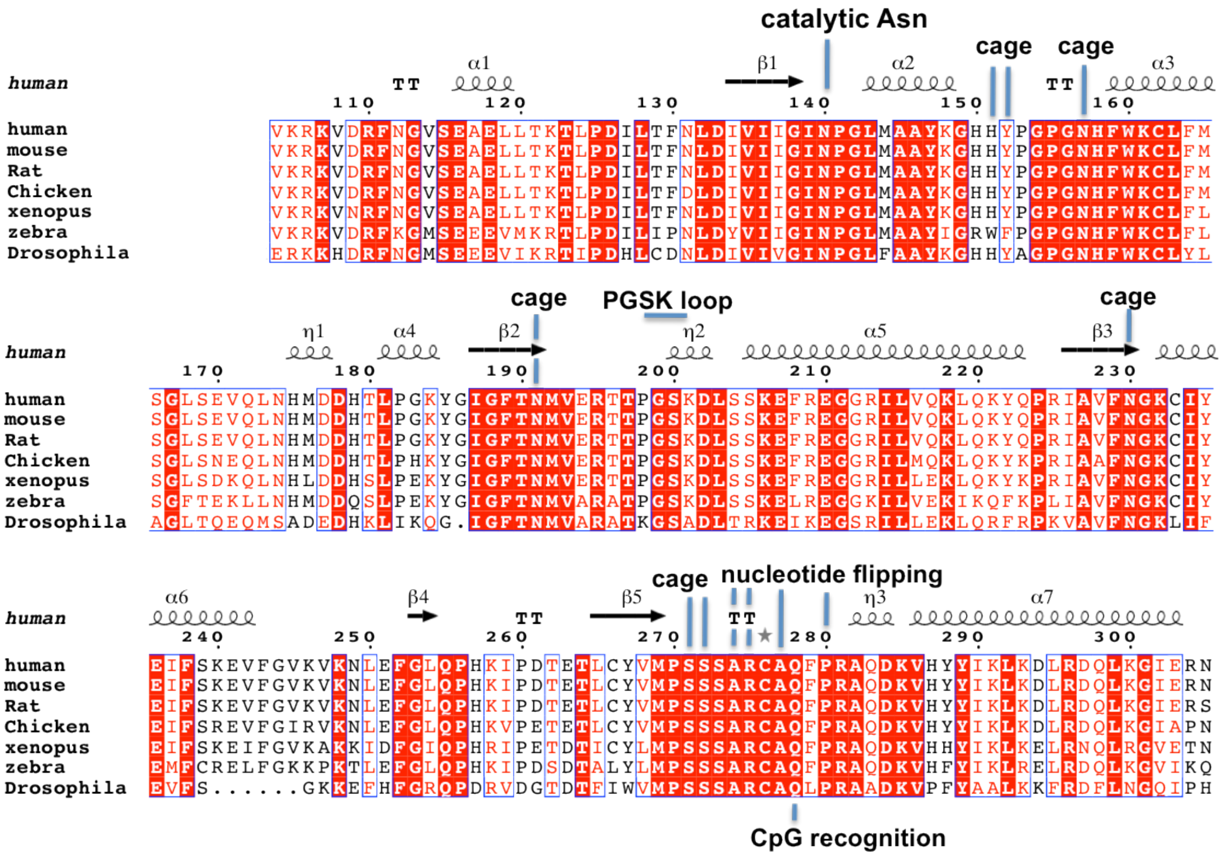

Supplementary Figure S4. Sequence alignment of the TDG family members.

Residues with white letter-on-red background are invariant among the seven sequences examined (human, NP_003202.3; mouse, NP_035691; Rat, EDM17058.1; zebra fish, NP_001018587; Xenopus, NP_001084290; Chicken, NP_990081; and Drosophila, NP_651925.1. Conserved cage residues, capable of making hydrogen-bonding interactions via proton donor or acceptor, are indicated.

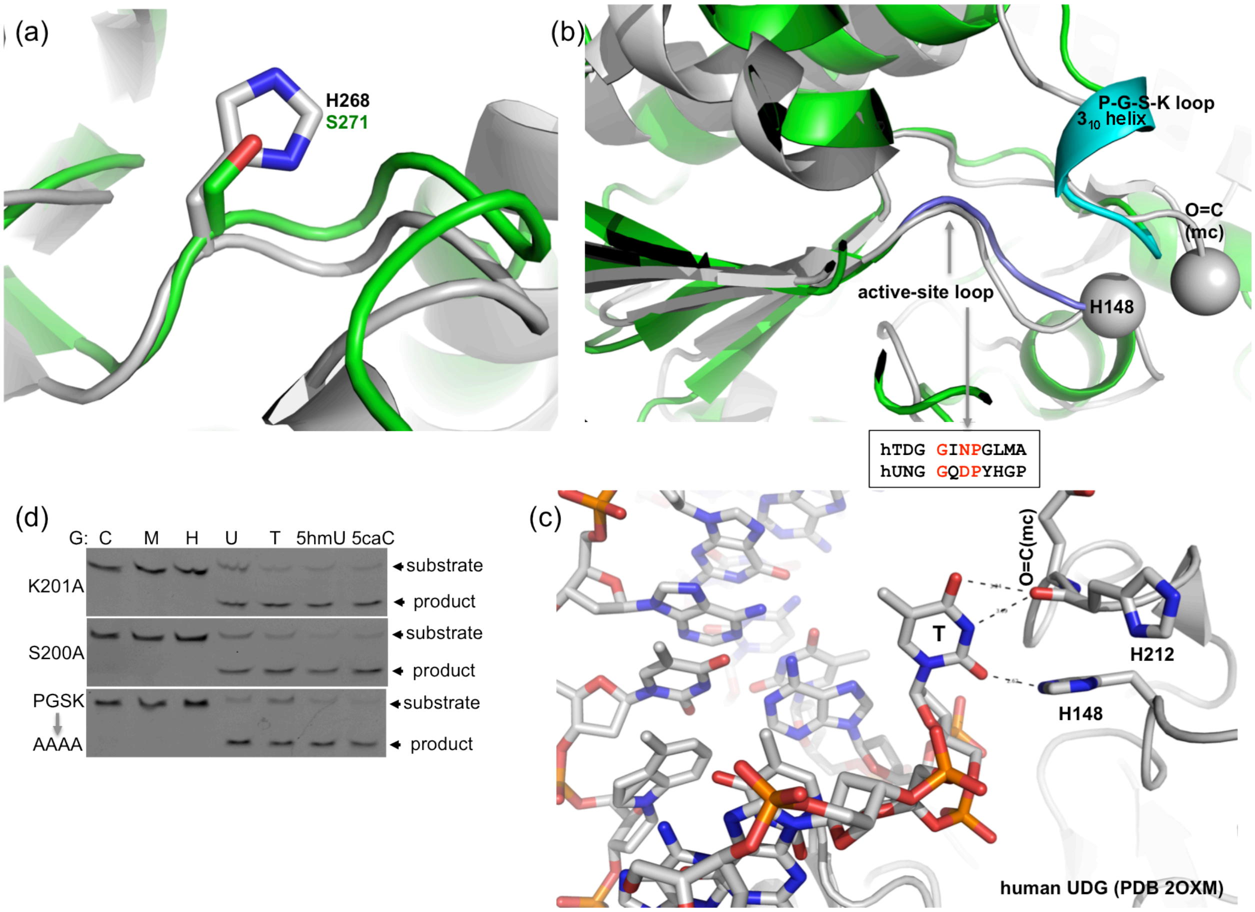

Supplementary Figure S5. Structural comparison of human TDG and human UNG

(a) Ser271 of hTDG and His268 of hUNG occupy similar positions in their respective active sites.

(b) Superimposition of hTDG (colored) and hUNG (grey) indicates that the P-G-S-K loop of hTDG is located in the corresponding position of the thymine-interacting loop of hUNG (involving the main chain carbonyl atom of His212). His148 of hUNG corresponds to Leu143 in hTDG.

(c) The structure of human UNG bound to DNA containing a thymine (PDB 2OXM). The thymine is rotated from the base stack by about 30°, which is only one-sixth of the 180° rotation required to fully flip uracil into the active site pocket. Interactions with the thymine base involve the side chain of His148 and the main chain carbonyl oxygen of His212 to the polar edge atoms, but not the methyl group of the thymine.

(d) The mutants of S200A (pXC1120), K201A (pXC1102) or PGSK to AAAA (pXC1123) do not affect TDG activity under the conditions tested: double stranded 32 bp oligonucleotides bearing a single CpG dinucleotide were incubated with equal amount of the mutant proteins of TDG catalytic domain at 37 °C for 30 minutes. In hUNG, it is the main chain atoms of the corresponding loop that interact with a partially flipped thymine. It remains possible that the specific side chain interactions may come from different locations in hTDG.

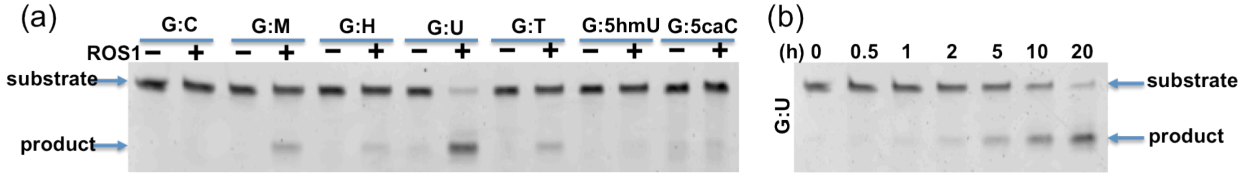

Supplementary Figure S6. Base excision activity of *Arabidopsis* ROS1 (REPRESSOR OF SILENCING 1)

(a) Double stranded 32-bp oligonucleotides bearing a single CpG dinucleotide (50 nM) were incubated for 20 h with equal molar ratio of purified ROS1 (see below) in a 20 µl reaction (50 mM Tris-HCl pH 8.0, 1 mM EDTA, 1 mM DTT and 0.1% BSA) at room temperature (approximately 22 °C). Reactions were stopped by adding 2 µl of 1 mg/ml Proteinase K and incubating at 50 °C on a heat block for 15 min. Negative controls were provided for each reaction in the absence of ROS1. (b) The time course of ROS1 activity on G:U substrate was measured under the same conditions as panel a. Under the conditions tested, we estimated ROS1 activity in the order of U > T ≈ 5mC (M) > 5hmC (H).

We followed the published purification procedure of ROS1 (47) with some modifications. Briefly, the full-length ROS1 (1393 residues) was expressed in BL21(DE3) *dcm*$^{-}$ Codon Plus cells (Stratagene) as a 6xHis fusion in a pET28a vector (Novagen). A 1 ml aliquot of the overnight culture was inoculated into 1 L of Luria-Bertani medium containing kanamycin (50 µg/ml) and chloramphenicol (25 µg/ml), and incubated at 37 °C, 250 rpm, until OD_{600} of approximately 0.1. The temperature was then lowered to 23 °C, and incubation continued at 250 rpm for approximately 90 min before adding 5 mM betaine, 5 mM Na-glutamate and 500 mM NaCl. When OD_{600} reached 0.7, protein expression was induced for 2 h with 1 mM isopropyl-1-thio-β-d-galactopyranoside. The stored pellet was thawed and re-suspended in the buffer (20 mM Tris-HCl pH 8.0, 500 mM NaCl, 5 % glycerol and 1 mM dithiothreitol) with 5 mM imidazole. Cells were lysed by sonication (10 min: 1 s on and 3 s off), and the lysate was clarified by centrifugation. The fusion protein was isolated on a nickel-charged HisTrap HP affinity column after two washes (150 ml with 5 mM imidazole and 50 ml with 100 mM imidazole, respectively) and eluted by 15 ml of 300 mM imidazole. The protein was loaded onto tandem HiTrap Q column and HiTrap SP column in the same buffer with 500 mM NaCl and eluted from HiTrap-SP column in a 30-ml step elution in the presence of 800 mM NaCl. Protein concentration was estimated by coomassie staining using Bovine Serum Albumin (BSA) as standard.

Supplementary Table S1. Data collection and refinement statistics (molecular replacement)

Data collection	hTDG (Residues 111-308)
DNA	G:5hmU (28 bp)
Space group	C2
Cell	(β=96.5°)
a (Å)	91.0
b (Å)	53.7
c (Å)	81.6
Beamline	APS 22-ID
Wavelength	1.00000 Å
Resolution (Å)*	39.16-2.49 (2.58-2.49)
R_{merge} *	0.081 (0.630)
<I/σI> *	25.3 (2.9)
Completeness (%) *	99.1 (93.6)
Redundancy *	10.2 (8.5)
Observed reflections	140,014
Unique reflections *	13,702 (1282)
**Refinement**	
Resolution (Å)	2.49
No. reflections	13,014
R_{work} / R_{free}	0.224/0.267
No. of atoms	
protein	1567
DNA	1149
water	38
B-factors ($Å^2$)	
protein	58.6
DNA	71.0
water	54.7
R.m.s. deviations	
Bond length (Å)	0.003
Bond angles (°)	0.7

* Highest resolution shell is shown in parenthesis.
